# Mesenchymal stem cells and their mitochondrial transfer: a double-edged sword

**DOI:** 10.1042/BSR20182417

**Published:** 2019-05-03

**Authors:** Cheng Li, Marco K.H. Cheung, Shuo Han, Zhao Zhang, Ling Chen, Junhui Chen, Hui Zeng, Jianxiang Qiu

**Affiliations:** 1 Guangzhou Women and Children’s Medical Center, Guangzhou Medical University, Guangzhou, China; 2 Stem Cell and Regenerative Medicine Consortium, the University of Hong Kong, Hong Kong, SAR, China; 3 Intervention and Cell Therapy Center, Peking University Shenzhen Hospital, Shenzhen, China; 4 Department of Orthopaedics, Peking University Shenzhen Hospital, Shenzhen, China

**Keywords:** mesenchymal stem cell, mitochondrial dysfunction, mitochondrial transfer

## Abstract

Mitochondrial dysfunction has been linked to many diseases including organ degeneration and cancer. Mesenchymal stem cells/stromal cells (MSCs) provide a valuable source for stem cell-based therapy and represent an emerging therapeutic approach for tissue regeneration. Increasing evidence suggests that MSCs can directly donate mitochondria to recover from cell injury and rescue mitochondrial damage-provoked tissue degeneration. Meanwhile, cancer cells and cancer stromal cells also cross-talk through mitochondrial exchange to regulate cancer metastasis. This review summarizes the research on MSCs and their mitochondrial transfer. It provides an overview of the biology, function, niches and signaling that play a role in tissue repair. It also highlights the pathologies of cancer growth and metastasis linked to mitochondrial exchange between cancer cells and surrounding stromal cells. It becomes evident that the function of MSC mitochondrial transfer is a double-edged sword. MSC mitochondrial transfer may be a pharmaceutical target for tissue repair and cancer therapy.

## Introduction

Mitochondria are well known as biological engines that generate energy. They decompose carbohydrates to produce adenosine triphosphate (ATP) through oxidative phosphorylation (OXPHOS), providing most of the energy needed for metabolism and movement in living organisms [1,2]. At the same time, mitochondria are also related to cellular stress responses including apoptosis and autophagy [1]. Specifically, in OXPHOS, respiratory complexes I and III generate reactive oxygen species (ROS), which modulates homeostatic signaling pathways in cellular growth and stress responses. However, ROS can also damage cell proteins, lipids and nucleic acids [1].

Mitochondrial dysfunction is one of the hallmarks of aging and is associated with a large proportion of human diseases [1,3]. ROS is one of the factors contributing to diseases associated with mitochondrial dysfunction, but its effects on cellular aging are conflicting [3,4]. One possible explanation is that ROS primarily exerts a compensatory response in adapting to stress, but when cellular damage accumulates in advanced age, excessive ROS levels alter a cell’s homeostatic properties to exacerbate age-related damage. Mitochondrial dysfunction can also be attributed to ROS-independent factors, including a breach of mitochondrial integrity [3] and defective mitochondrial quality control [4]. Although mutations in mitochondrial DNA (mtDNA) or nuclear genes encoding mitochondrial proteins give rise to inherited mitochondrial diseases, mitochondrial dysfunction has also been associated with degenerative diseases such as Parkinson’s disease, Alzheimer’s disease and type 2 diabetes [5].

Mesenchymal stem cells/stromal cells (MSCs) are generally derived from bone marrow [6] and adipose tissue [7], or from induced pluripotent stem cells [8,9]. The therapeutic function of MSCs is achieved mainly through paracrine, differentiation and anti-inflammatory actions [10–13]. Studies have also demonstrated that MSCs have the potential to replace the defective mitochondria or compensate for their malfunction through mitochondrial transfer between MSCs and aging cells. MSCs have become popular in cell-based therapy following their promising results in animal studies and clinical trials. Recently, Japan approved the first use of autologous adipose tissue-derived MSCs in treating Alzheimer’s disease. Seemingly, the utilization of mitochondrial transfer in MSCs has a bright future in cell-based therapy.

However, mitochondrial transfer also plays a positive role in tumor growth. Transferred mitochondria produce detrimental effects to health as they sustain the growth of cancer cells by maintaining macromolecular biosynthesis and cytoplasmic pools of NAD^+^ for high glycolytic flux [14]. Worse still, PGC-1α expression in invasive cancer cells can enhance mitochondrial biogenesis and respiration, both of which are important for cell motility and metastasis [15]. Similar to normal cells, cancer cells require an optimal level of ROS to avoid excessive oxidative damage and consequent cell death [14].

In brief, mitochondrial transfer is a double-edged sword. In this mini-review, we aim to illustrate the beneficial and harmful effects of MSC mitochondrial transfer, as well as to discuss opportunities for future stem cell research.

### Mitochondrial transfer mediates tissue repair

**Figure 1:**
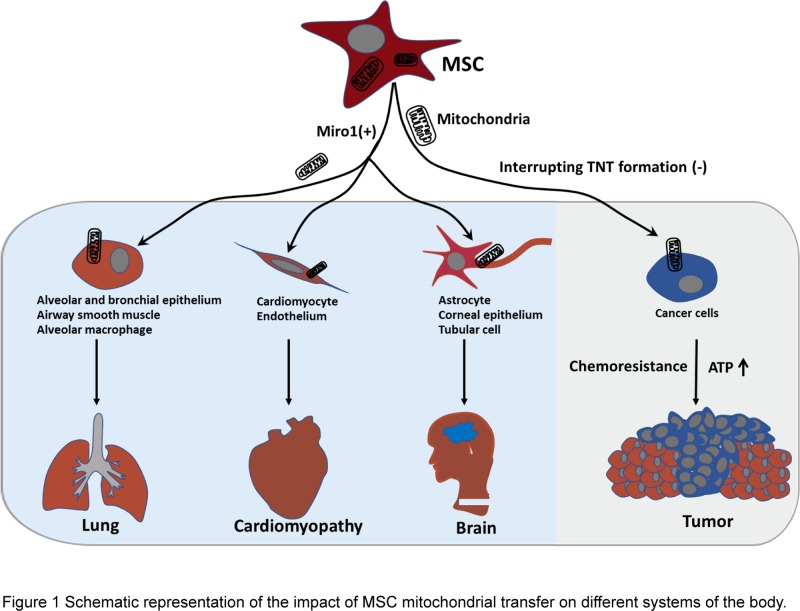
Schematic representation of the impact of MSC mitochondrial transfer on different systems of the body MSC mitochondrial transfer promotes the repair of lungs in the respiratory system, alleviates the symptoms of cardiomyopathy in the circulatory system, and relieves the symptoms of neurological diseases, such as Alzheimer’s disease, by increasing mitochondrial membrane potential, recovering aerobic respiration, reducing apoptosis, or ameliorating inflammation. MSC mitochondrial transfer also promotes tumor growth by enhancing OXPHOS, increasing ATP levels or resulting in greater chemoresistance. Up-regulation of Miro1 expression (Miro+) increases the efficiency of MSC mitochondrial transfer, and then promotes tissue repair. Selectively blocking of tunneling nanotube (TNT) formation can inhibit tumor growth promoted by MSC mitochondrial transfer.

Numerous studies have demonstrated that transfer of mitochondria between MSCs and damaged tissue is a key mechanism for tissue repair. The first description of the transfer was the acquisition of functional mitochondria in A549 ρ° cells with mtDNA deletions or defects when they were co-cultured with human MSCs [16]. The recovery of function was demonstrated by mitochondrial activities such as increased oxygen consumption, membrane potential and intracellular ATP levels. Since the first success, MSC mitochondrial transfer has been widely studied in the cardiovascular, respiratory, neurological and renal systems (Figure 1).

#### Mitochondrial transfer from MSCs to cardiovascular system

Studies have shown that it is possible to transfer human bone marrow-derived MSC (BM-MSC) mitochondria to animal embryonic and adult cardiomyocytes [17–19]. Co-culture of BM-MSCs and ischemic rat cardiomyocytes has been shown to increase mitochondrial membrane potential in the latter cells [17]. Furthermore, stem cell mitochondrial transfer plays a significant role in cardiomyocyte reprogramming. BM-MSCs have been shown to be able to reprogram mature cardiomyocytes to progenitor-like cells with increased gene expression in early cardiac commitment through partial cell fusion [18]. In addition, BM-MSCs have been studied in ischemic vascular diseases and been shown to rescue human umbilical vein endothelial cells (HUVECs) with dysfunctional mitochondria [20]. Aerobic respiration was recovered, and apoptosis of ischemic endothelial cells (ECs) was reduced following mitochondrial transfer from BM-MSCs to HUVECs. Although human induced pluripotent stem cell-derived MSCs (iPSC-MSCs) and BM-MSCs can both deliver mitochondria to mice cardiomyocytes with doxorubicin-induced damage, iPSC-MSCs have demostrated a higher transfer efficiency than BM-MSCs [21].

Intriguingly, mitochondria are not confined to a unidirectional transfer from MSCs to injured cells. In co-culture of human BM-MSCs and coronary artery vascular smooth muscle cells (VSMCs), mitochondrial transfer has been demonstrated to be bidirectional with no preferential direction of movement [22]. Transfer from VSMCs to MSCs increased MSC proliferation, but not differentiation, and this effect was lost if VSMCs had dysfunctional mitochondria due to mtDNA defects. Further experiments showed that human cardiomyocytes and HUVECs challenged by hydrogen peroxide could deliver mitochondria to MSCs as a danger signal [23]. MSCs underwent autophagy of foreign mitochondria, and degraded products increased the amount of heme oxygenase-1 in the cytoplasm, which then stimulated biogenesis of MSC mitochondria. The mitochondrial transfer efficiency of MSCs increased to rescue the injured cells from apoptosis. Similar to hydrogen peroxide, MSCs were able to recover doxorubicin-damaged cells and produced ROS was involved in heme oxygenase-1 stimulation and mitochondrial biogenesis.

#### Mitochondrial transfer from MSCs to respiratory system

In a study of acute lung injury induced by *Escherichia coli* lipopolysaccharide, mouse BM-MSCs reduced leukocyte numbers, restored surfactant secretion and enhanced survival of mouse lungs [24]. Mitochondrial transfer from BM-MSCs to alveolar epithelium was revealed by fluorescence microscopy and led to an increased alveolar ATP level. Additionally, iPSC-MSCs attenuated the severity of alveolar destruction and fibrosis in rats with lung damage induced by cigarette smoke, mimicking chronic obstructive pulmonary diseases [25]. Mitochondria were shown to be delivered from iPSC-MSCs to damaged bronchial epithelial cells. iPSC-MSCs are also capable of delivering mitochondria to airway smooth muscle cells [26], especially in the cigarette smoke medium, to ameliorate inflammation and airway hyperresponsiveness in human lung cells and mouse lungs. Moreover, mitochondrial transfer from iPSC-MSCs to ECs through tunneling nanotubes (TNTs) alleviated asthma inflammation in mice [27]. Apart from offering mitochondria to epithelial and muscle cells, MSCs have a role in the respiratory immune system. The donation of mitochondria to macrophages *in vivo* and *in vitro* has been shown to promote their phagocytic capacity and suppress pro-inflammatory cytokine secretions in an acute respiratory distress syndrome environment [28,29]. The discovery of active mitochondrial transfer for antimicrobial effects of MSCs in the acute respiratory distress syndrome animal model induced by *E. coli* pneumonia [28] may warrant further investigations in other infections.

#### Mitochondrial transfer from MSCs to neurological and renal systems

Astrocytes and neuron-like pheochromocytoma cells have been shown to receive mitochondria from MSCs in co-cultivation, as revealed by fluorescence microscopy [30]. Mitochondria transfer from MSCs to astrocytes was stimulated by oxidative stress with an increased ROS level, and the transfer to neuron-like cells recovered cellular aerobic respiration and proliferation. The stimulation of oxidative stress in transfer effectiveness has also been demonstrated in corneal epithelial cells [31]. Efficient mitochondria transfer from MSCs is essential for corneal protection and wound healing in damaged epithelial cells. Another experiment showed that MSCs could donate mitochondria to rat renal tubular cells, and rat renal tubular cells could also, to a lesser extent, transfer their mitochondria back to MSCs [32].

### Transfer of mitochondria supports tumor progression

Inflammation is an essential part of the malignant microenvironment and contributes to tumor progression [33]. In tumor-related inflammation, chemokines are key players and will attract BM-MSCs to the site of inflammation [34–36]. To establish a favorable tumor microenvironment, BM-MSCs may differentiate into cancer-associated fibroblasts, play an immunomodulatory function and therefore promote cancer cell growth and migration [37,38].

Although mitochondrial transfer from MSCs to damaged cells has produced encouraging results in tissue repair, the effects on cancer cells are not desirable (Figure 1). Tumor growth and motility require mitochondria. MSCs can deliver mitochondria to breast cancer cells and glioblastoma stem cells [39,40]. Therefore, with the increased OXPHOS and ATP production, cell proliferation and invasion ability are enhanced after receiving MSC mitochondria [39]. In breast cancer cells, while the fusion between endogenous and exogenous mitochondria was not observed, a small amount of mtDNA from MSCs was found. The mechanisms of how transferred MSC mitochondria work, whether independently or not, remain elusive and a subject for further studies.

#### Mitochondrial transfer promotes chemoresistance of breast cancer cells

Acquisition of mitochondria also results in greater chemoresistance of breast cancer cells to doxorubicin [41]. Surprisingly, mitochondria have been found to be transferred from ECs, instead of MSCs, to cancer cells. Transfer of cytoplasmic content, but not mitochondria, has been evidenced from MSCs to cancer cells. P-glycoprotein 1 expression, which may confer multiple drug resistance in cells, has been reported absent from both cancer cells and ECs. Even in the tri-culture of cancer cells, ECs and MSCs, MSC mitochondria were not transported, while both MSCs and cancer cells received EC mitochondria. Although MSCs were capable of delivering mitochondria to damaged ECs [20,23], the complex interaction of mitochondria transfer between MSCs and multiple cell types is yet to be elucidated.

#### Mitochondrial transfer promotes proliferation and chemoresistance of leukemia

In addition to solid cancers, mitochondrial transfer may also worsen the outcome of hematological cancers. When acute myelogenous leukemia (AML) cells received mitochondria from BM-MSCs, they demonstrated increased OXPHOS and ATP production, as well as chemoresistance to cytarabine [42]. Moreover, mitochondrial transfer from MSCs and subsequent incorporation into AML cells was stimulated by treatment with cytarabine. Other chemotherapeutic drugs including etoposide and doxorubicin, but not vincristine, can also trigger incorporation of mitochondria. A remarkable finding has been that such transfer was enhanced by ROS [43], similar to the study in doxorubicin-damaged cells [23]. ROS was revealed to be derived from NADPH oxidase-2. For T cell acute lymphoblastic leukemia (T-ALL) cells, mitochondrial transfer has been shown to be bidirectional between MSCs and T-ALL cells [44]. In contrast with AML cells, chemotherapeutic agents cytarabine and methotrexate stimulated mitochondrial transfer from T-ALL cells to MSCs, with only a few obtained from T-ALL cells. The ROS levels in T-ALL cells were hence reduced to enable their proliferation and survival. T-ALL cell chemoresistance was also enhanced. The disparity between AML and ALL cells might be explained by different adhesive properties, metabolic states, need for OXPHOS and glycolysis and hence opposite direction of mitochondrial transfer.

### Future directions in MSC mitochondrial transfer

#### Elucidation of regulation of mitochondrial transfer

**Figure 2:**
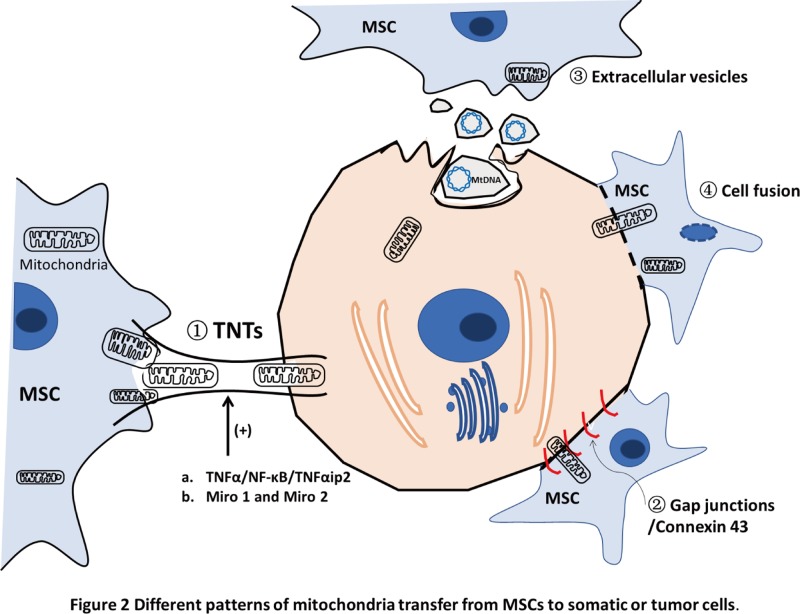
Different patterns of mitochondria transfer from MSCs to somatic or tumor cells The mechanisms include transfer through intracellular TNTs, extracellular vesicles (such as exosomes), cell fusion and gap junctions. TNT formation can be regulated by TNFα/NF-κB/TNFαip2 signaling pathway and promoted by high levels of Miro1 and Miro2.

The main mechanisms of intercellular mitochondrial transfer adopted so far are shown in Figure 2 and expounded as follows:

1. At present, the theory accepted by most scholars is the formation of TNTs between the recipient cells and the donor cells with complete transfer of mitochondria achieved through active transport [21,23,25–28,31]. Several factors have been found associated with mitochondrial transfer along TNTs. Studies have shown that exercise-binding protein complexes regulate mitochondrial transport and homeostasis in TNTs. Miro 1 and Miro 2 are two Rho-GTPases linking mitochondria to kinesin and other accessory proteins, which allows mitochondria to move along the TNTs connecting two cells [45]. With higher expression of Miro 1, MSCs showed a more efficient mitochondrial transfer in cardiomyopathy [21]. Down-regulation of Miro1 expression inhibited TNTs formation and prevented the transfer of mitochondria from MSCs to ECs, demonstrating the important role of Miro1 in the transfer of mitochondria through TNTs [46]. Tumor necrosis factor α (TNFα) induces the formation of TNTs in cardiomyocytes and corneal epithelial cells by increasing TNFαIP2 expression [21,31]. NF-κB is a transcription factor in a conserved cascade pathway involved in diverse processes, such as cytokine expression, stress regulation, cell division and transformation by targeting on different genes [47]. Studies have shown that TNFα promoted the levels of NF-κB subunits p-IκB and p-P65, and TNFαip2, while the NF-κB inhibitor SC-514 significantly attenuated the levels of p-P65 and TNFαip2, and subsequently abolished the formation of TNTs [21,31]. These results suggest the regulation function of TNFα/NF-κB/TNFαip2 signaling pathway in TNT formation. At the same time, TNTs may be associated with calcium signaling [48]. The physiological role of TNT-dependent electrical coupling is synchronized with cell migration activity, and calcium is the major ion that conveys this synchronizing effect to regulate proliferation, migration and differentiation in neurons [49].
2. Gap junctions are also shown to mediate mitochondria transfer of MSCs. Connexin 43-containing gap junction channels has been particularly well observed in a mouse model of acute lung injury *in vivo* and deliver mitochondria in microvesicles to the epithelia from bone marrow-derived stromal cells [24]. Blocking connexin-43 gap junction formation had no effect on cytoplasmic transfer, but reduced mitochondrial transfer [50].
3. mtDNA from MSCs is transferred via extracellular vesicles. It has been recently demonstrated that migration of mtDNA can take place via exosomes [51]. Extracellular vesicles from human MSCs have shown a therapeutic effect on severe pneumonia in mice, possibly by the transfer of mitochondria or the mRNA of key mitochondrial genes [52]. Another study indicates that MSCs could release mitochondrial particles in extracellular vesicles, which carried mtDNA and were taken up by macrophages to enhance their OXPHOS activity [53]. Through extracellular vesicles, such as exosomes, mtDNA can enter into other cells.
4. It has also been reported that mitochondria transfer can be achieved by selective loss of donor nuclei after complete cell fusion [54].

#### Exploring mechanisms of directionality of mitochondrial transfer

Generally, mitochondrial transfer is directional, which is determined by some factors. (i) Several studies have demonstrated that directionality of mitochondrial transfer is associated with cytoskeletal microfilaments in microvesicles of connected tubular structures [24,55,56]. Plotnikov et al. [19] esablished that cytoplasm transfer was in a non-directional manner between cells, while mitochondrial transfer could only be from BM-MSCs to cardiomyocytes. They observed the classical submicroscopic structure and cytoskeletal structure of mitochondria in the microbubbles connected to the pipeline structure, suggesting that the cytoskeletal microfilament may be an important element guiding the one-way transport of microbubbles. (ii) Another study suggested that receptor cell damage may promote unidirectional transfer of mitochondria from MSCs to damaged cells [23]. (iii) Recent studies have shown that some of the damage or apoptotic factors (such as phosphatidylserine) expressed on the surface of damaged cells can selectively aggregate and guide the formation of connecting ducts [57]. As bidirectional mitochondrial transfer has occasionally been observed [22,23,44], the mechanism about the directionality of mitochondrial transfer remains to be further elucidated.

#### Potential targeted therapy against mitochondrial transfer in cancer

Mitochondrial biogenesis and respiration are critical for tumor cell growth, survival and metastasis [15]. Dong et al. [58] have shown that mtDNA is critical for the production of tumors in mice. In the absence of mtDNA, metastatic melanoma cells with defective respiratory function could not form tumors. After obtaining complete mitochondria and mtDNA by mitochondrial transfer, the respiratory function of the cells could be restored [58]. It is demonstrated that selectively blocking TNT formation prevents mitochondrial metastasis, and thereby inhibits tumor growth [59]. Studies in hormonal therapy-resistant metastatic breast cancer have also shown that horizontal transfer of mtDNA via extracellular vesicles plays a role in an exit from dormancy of cancer stem-like cells and leads to endocrine therapy resistance [60]. Inhibition mitochondrial function in epithelial cancer cells could overcome the anti-estrogen resistance in breast cancer [61]. Many drugs target and alter mitochondrial function during cancer treatment [62]. Some studies have shown that mitochondria can be used to transport some pro-oxidant anticancer compounds, such as vitamin E analogs and phenylethyl isothiocyanate, which can induce apoptosis in cancer cells by producing ROS [63]. Mitochondria-targeted vitamin E succinate has been reported to effectively induce apoptosis in human breast cancer cell line MCF-7 [63]. In view of the multiple metabolic pathways of cancer cells, mitochondrial targeting to treat cancer remains to be further studied.

#### Potential therapeutic use of mitochondrial transfer in mitochondria-related diseases

Myocardial ischemia and reperfusion results in severe mitochondrial loss that can lead to ischemic necrosis of cardiomyocytes [64]. Clinical trials have demonstrated that autologous mitochondria can be extracted from the patient’s muscle for autologous treatment [65,66]. Intravenous mitochondria treatment of mitochondrial diseases has been tried in multiple organs. When exogenous mitochondria are administered intravenously to mice, they can be rapidly distributed to multiple tissues and organs. Therefore, mitochondrial transfer can be used to treat mitochondria-related diseases involving multi-tissue lesions. Intravenous injection of exogenous mitochondria into a mouse model of Parkinson’s disease has been shown to significantly improve the symptoms of Parkinson’s disease. The mechanism is related to increased electron transport chain activity, reduced the level of reactive oxygen free radicals and prevention of apoptosis and necrosis [67].

## Conclusions

Mitochondrial transfer is a ubiquitous phenomenon that is associated with various physiological and pathological activities of the body. Therefore, mitochondrial treatment through mitochondrial transfer has potentially broad applications. We can effectively treat with MSCs by mitochondrial transfer to restore normal physiological functions of cells and recover patients from diseased condition. Meanwhile, inhibition of mitochondrial transfer from stromal cells to cancer cells may serve a potential therapeutic target.

## Abbreviations

AML: acute myelogenous leukemia
ATP: adenosine triphosphate
BM-MSC: bone marrow-derived MSC
EC: endothelial cell
HUVEC: human umbilical vein endothelial cell
iPSC-MSC: induced pluripotent stem cell-derived MSC
mtDNA: mitochondrial DNA
MSC: mesenchymal stem cell/stromal cell
OXPHOS: oxidative phosphorylation
ROS: reactive oxygen species
TNFα: tumor necrosis factor α
TNT: tunneling nanotube
T-ALL: T cell acute lymphoblastic leukemia
VSMC: vascular smooth muscle cell
